# Exploring the Potential of the CamAPS FX Advanced Hybrid Closed-Loop (AHCL) System in the Hospital Setting: From Pregnancy to a Postpartum Complicated by Infective Endocarditis of the Tricuspid Valve

**DOI:** 10.7759/cureus.96075

**Published:** 2025-11-04

**Authors:** Silvia Galasso, Sara Piccini, Viviana Casarsa, Sandra Agus, Andrea Da Porto

**Affiliations:** 1 Diabetes and Metabolism Unit, Azienda Sanitaria Universitaria Friuli Centrale (ASUFC), Udine, ITA

**Keywords:** diabetes type 1, hacl, hospitalization, insulin pump, postpartum, pregnancy

## Abstract

Managing glycemic control in type 1 diabetes during pregnancy is still very complex and extremely important to reduce maternal and fetal risks. We present the case of a 36-year-old woman with type 1 diabetes mellitus managed throughout her pregnancy with an advanced hybrid closed-loop (AHCL) CamAPS FX. Glycemic control during pregnancy was optimal, with a time in range (64-140 mg/dL) in the second and third trimesters > 75%. No serious episodes of hypoglycemia or ketoacidosis occurred. A vaginal delivery occurred at the 39th week, with a favorable neonatal outcome (birth weight 3,418 g, Apgar score 8/9). The uniqueness of this clinical case is related to the fact that the patient experienced several postpartum complications: hospitalization for tricuspid valve endocarditis, which initially manifested with pulmonary embolism. The postpartum period, characterized by breastfeeding, changes in sleep-wake rhythm, newborn care, infectious disease, antibiotics, and hospitalization, was managed by the algorithm. The insulin pump was never interrupted and continued with the support of the diabetes team, achieving optimal glycemic control throughout the postpartum period until the acute condition resolved with a time in range (70-180 mg/dL) of 88%. The CamAPS system in our case has therefore proven to be effective, safe, and adaptable during pregnancy, the postpartum period, and hospitalization during the acute phase of the disease.

## Introduction

The achievement of optimal glycemic control during pregnancy remains challenging for patients with type 1 diabetes. Strict glycemic control is the optimal management for pre-existing type 1 diabetes in pregnancy, as HbA1c levels and time in range (TIR) are associated with the risk of maternal and fetal complications. Advanced hybrid closed-loop (AHCL) therapy can improve glycemic control in pregnant women with type 1 diabetes. The use of AHCL systems has recently been shown to be beneficial also in the postpartum period, preventing the worsening of glucose control observed with standard insulin therapy [1]. The use of AHCL in the case of postpartum complications or rehospitalization can be challenging due to the rapid changes in insulin needs.

AHCL insulin pumps are systems composed of continuous glucose monitoring (CGM), an insulin pump, and an algorithm that automatically adjusts insulin delivery based on blood glucose readings, while meal boluses remain managed by the user. The CamAPS FX algorithm was the first AHCL system approved for use during pregnancy and, at the time of our case report, the only one available. The CamAPS FX algorithm uses Model Predictive Control (MPC), a control system that employs predictive modeling to optimize insulin delivery. The algorithm predicts future blood glucose levels based on the current state, active insulin (insulin on board (IOB)), planned boluses, and carbohydrate (CHO) intake. User-entered parameters include body weight and total daily insulin dose (TDD). The algorithm automatically estimates additional parameters, such as insulin sensitivity and insulin action time, and continuously updates them to reflect daily variations in blood glucose, insulin requirements, and individual responses. Different blood glucose targets can be set; in addition, two special modes are available to adjust the algorithm’s aggressiveness: "Ease-off" (a more conservative mode, useful during exercise) and "Boost" (a more aggressive mode, suitable in conditions of increased insulin resistance) [2]. We report the case of a type 1 diabetes patient using the CamAPS FX system during pregnancy and an early postpartum hospitalization.

## Case presentation

A 36-year-old female patient was followed at our outpatient clinic for type 1 diabetes. She has been affected by diabetes since the age of 17. She had been using an insulin pump since 2014 and transitioned to the CamAPS FX system in 2023 for pregnancy planning. Her medical history was also notable for a heterozygous factor V Leiden mutation. Glycemic control before pregnancy was good, with an HbA1c level of 5%. She had no diabetes complications. Her body mass index was within the normal range at 20 kg/m^2^. The patient conceived through medically assisted reproduction (in vitro fertilization).

The CamAPS settings during the first trimester of pregnancy were the following: the insulin-to-CHO (IC) ratio was 1:10 for breakfast, 1:12 for lunch, and 1:13 for dinner. Glucose targets were 95 and 85 mg/dL during daytime and nighttime, respectively. In the second trimester, the IC ratio was set at 1:9 for breakfast, 1:11 for lunch, and 1:11 for dinner, and glucose targets were 91 mg/dL during the day and 81 mg/dL during the night. In the third trimester, IC ratios were 1:6 for breakfast, 1:10 for lunch, and 1:10 for dinner (Table 1).

**Table 1:** A1c levels and insulin doses at the end of each trimester of pregnancy N/A: not applicable; CHO: carbohydrate

Parameters	1st trimester	2nd trimester	3rd trimester	Recommended pregnancy-specific target range
A1c%	5.1	4.7	5	<6.5
Insulin-to-CHO ratio	1:10 for breakfast, 1:12 for lunch, 1:13 for dinner	1:9 for breakfast, 1:11 for lunch, 1:11 for dinner	1:6 for breakfast, 1:10 for lunch, 1:10 for dinner	N/A
Insulin sensitivity factor	80	60	50	N/A
Total daily insulin dose (IU)	28.4	33.4	49	N/A
Glucose target day (mg/dL)	95	91	91	<90 fasting; <130 after meal
Glucose target night (mg/dL)	85	81	81	<90 fasting; <130 after meal

Throughout pregnancy, blood glucose parameters remained within the desirable limits recommended by the guidelines. In the first trimester, HbA1c was 5.1%, TIR (64-140 mg/dL) 73%, time below range (TBR) 2%, time above range (TAR) 25%, and mean total daily dose of insulin 28.4 units. In the second trimester, HbA1c was 4.7%, TIR 77%, TBR 3%, TAR 20%, and mean total daily dose 33.4 units. In the third trimester, HbA1c was 5%, TIR 75%, TBR 3%, TAR 22%, and mean total daily dose 49 units (Tables 1, 2).

**Table 2:** Sensor glucose metrics during each trimester of pregnancy using recommended pregnancy-specific target ranges N/A: not applicable; CV: coefficient of variation

Trimester	Mean sensor glucose (mg/dL)	Sensor glucose CV (%)	Time < 54 mg/dL (%)	Time < 63 mg/dL (%)	Time in range (64–140 mg/dL) (%)	Time 141–250 mg/dL (%)	Time > 250 mg/dL (%)
1	119	32.2	1	1	73	24	1
2	112	31.3	1	2	77	20	0
3	115	31	1	2	75	22	0
Recommended pregnancy-specific target range	N/A	<36	<1	<4	>70	<25	0

**Figure 1:**
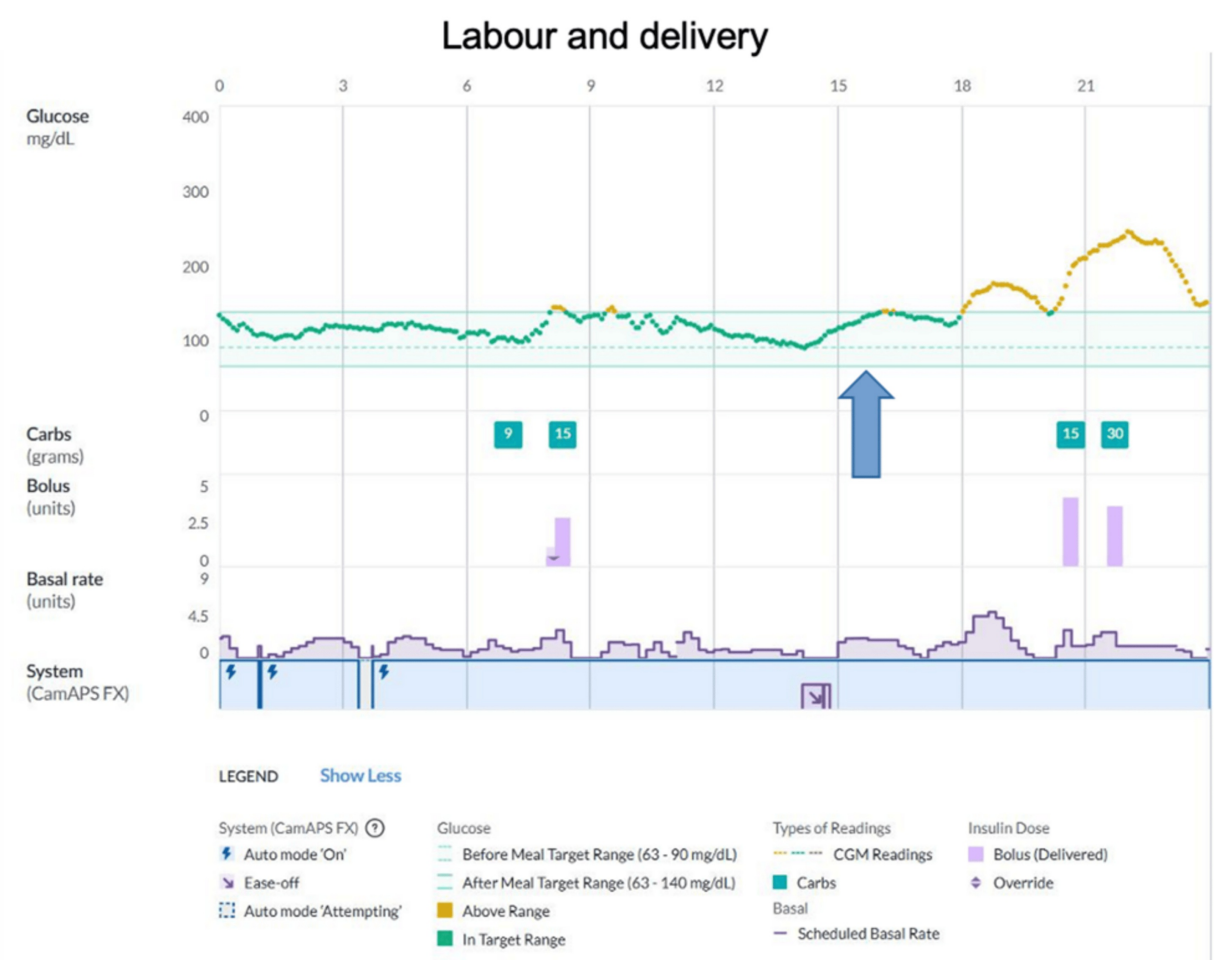
Labor and delivery CGM: continuous glucose monitoring

Overall weight gain was 18 kg. During pregnancy, she was also treated with low-molecular-weight heparin (LMWH) 6,000 IU daily and lysine acetylsalicylate 150 mg daily. Fetal growth during pregnancy was normal. In the second trimester, the following measurements were recorded: abdominal circumference 32nd percentile, head circumference 38th percentile, and estimated fetal weight 30th percentile. In the third trimester, the abdominal circumference was in the 84th percentile, the estimated fetal weight was in the 66th percentile, and the amniotic fluid was normal. Labor was induced at 39 weeks of gestation. An operative vaginal delivery was performed. A blood loss of 600 mL was reported; no transfusion was needed. LMWH was discontinued for two days peripartum. The newborn baby was a healthy male, measuring 3,418 g in weight and 50 cm in length, with an Apgar score of 8/9. The patient continued to utilize the CamAPS algorithm throughout labor. On the day of delivery, the CamAPS settings were as follows: target 110 mg/dL, insulin sensitivity factor 1:100 per unit, and IC ratios 1:12 for breakfast, 1:20 for lunch, and 1:20 for dinner. CGM metrics were TIR 74%, TAR 28%, TBR 0%, mean blood glucose 137 mg/dL, and SD 39 mg/dL. She used the ease-off mode only for about one hour during the active labor phase (Figure 1).

In the postpartum period, CamAPS settings were maintained as they were during labor. On the fourth day postpartum, both the mother and the infant were discharged from the hospital. The patient was instructed to continue receiving enoxaparin 4,000 UI daily upon discharge. Two days after being discharged from the gynecology ward, on the eighth day after delivery, the patient exhibited symptoms of tachycardia, dyspnea, chest pain, and fever, prompting her to seek treatment at the emergency room.

**Figure 2:**
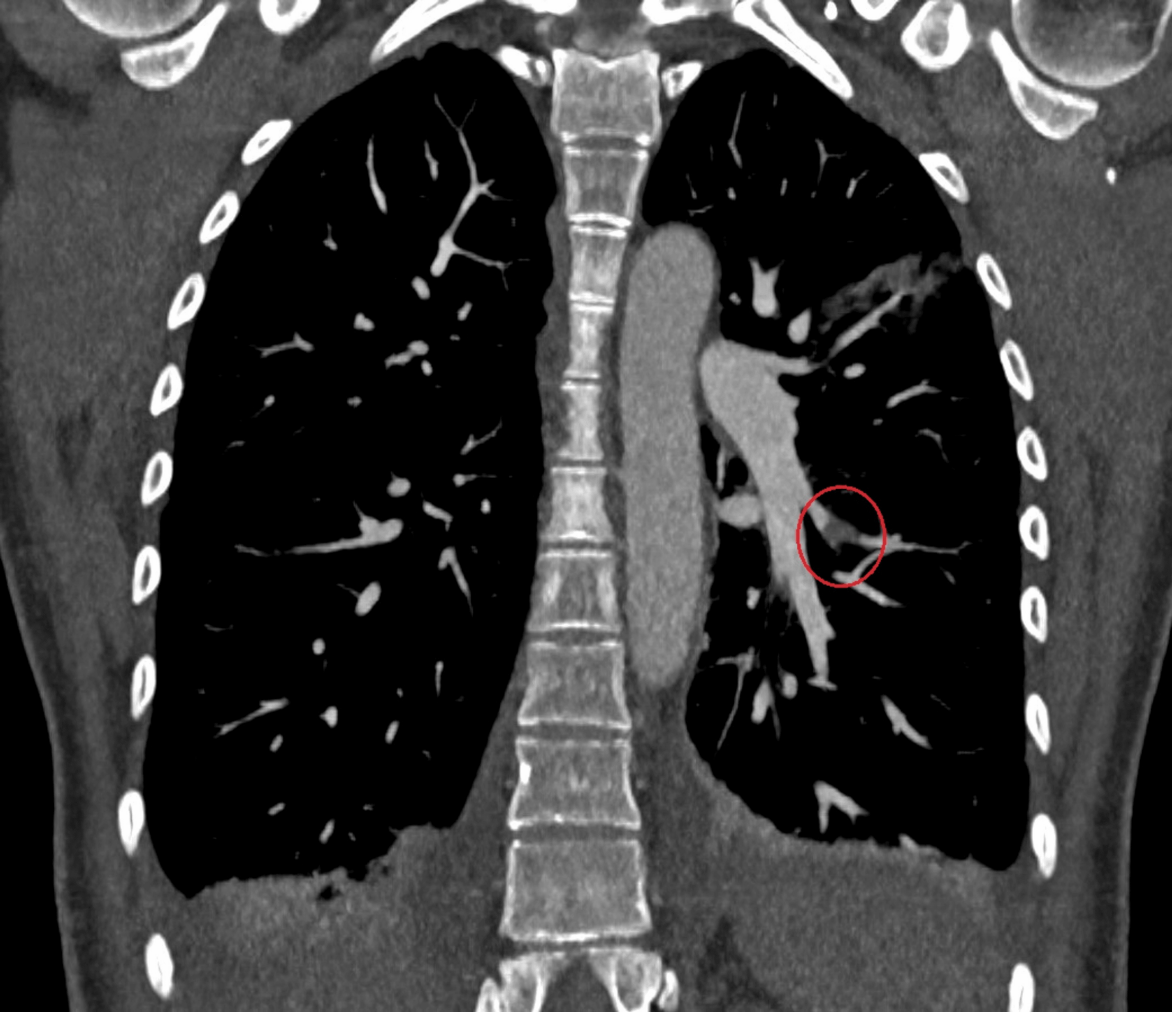
CT scan coronal plane

**Figure 3:**
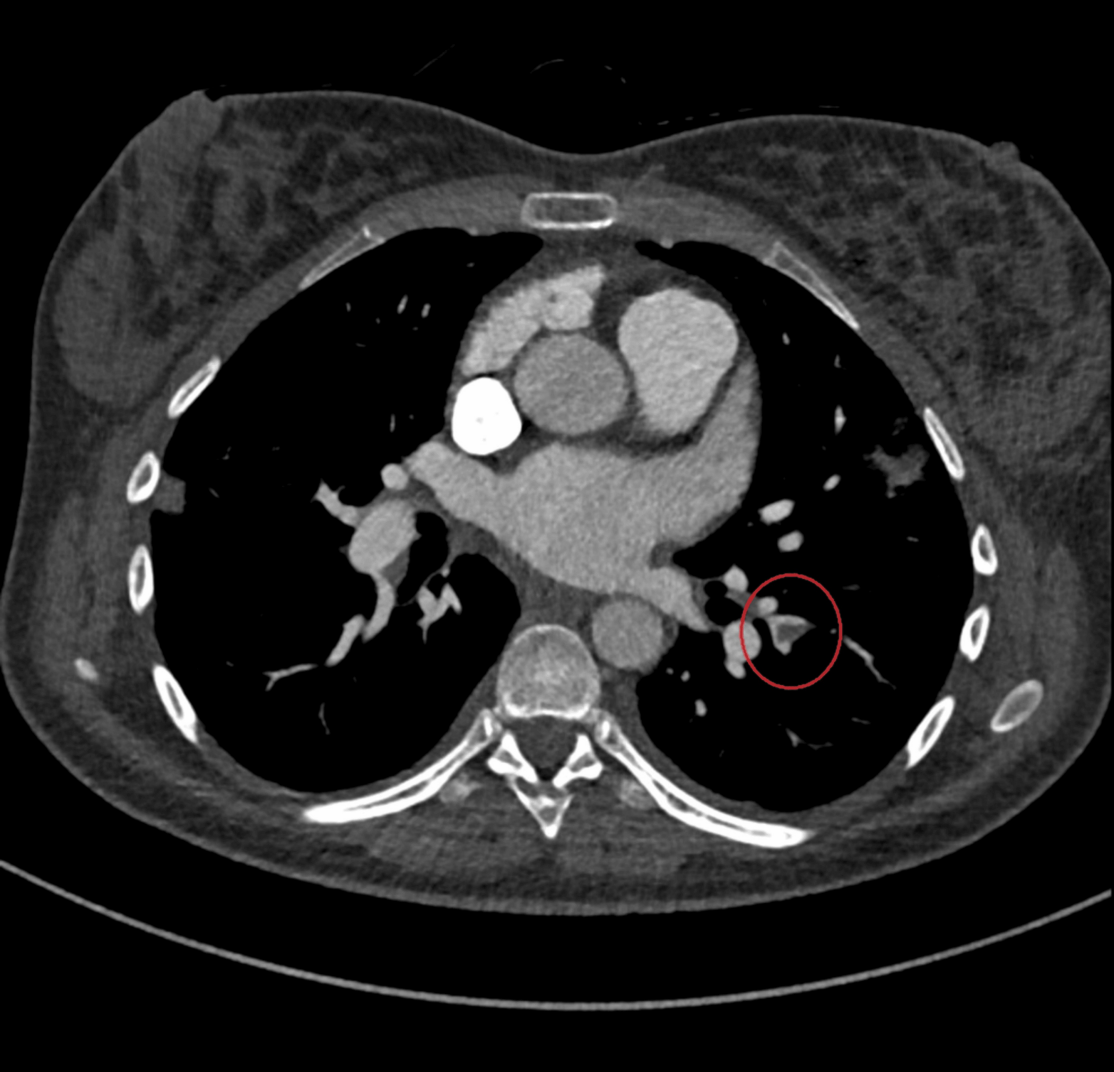
CT scan axial plane

Diagnostic possibilities were considered and ruled out. Endometritis was excluded due to the absence of abdominal pain and the presence of respiratory symptoms. Other potential diagnoses included pneumonia or pulmonary embolism, given the recent delivery. At the time of admission, the likelihood of pulmonary embolism was high, given the patient's recent delivery and medical history. A CT scan confirmed the suspicion of a pulmonary embolism with ground-glass areas compatible with infected pulmonary infarctions. A Doppler ultrasound of the lower limbs revealed no evidence of deep vein thrombosis (Figures 2, 3).

Consequently, she was admitted to the hospital and started on anticoagulant therapy (fondaparinux 7.5 mg QD, compatible with breastfeeding) and antibiotic therapy with amoxicillin/clavulanate. In the event of a fever spike (temperature > 38°C), blood cultures were collected. The results indicated the presence of gram-positive cocci, prompting a modification in the antibiotic therapy regimen. The treatment was transitioned to a combination of piperacillin/tazobactam and daptomycin. Subsequent blood culture results yielded the identification of *Staphylococcus aureus*, mecA gene negative. An Infectious Diseases consultation was subsequently requested, and according to the most recent version of the Duke criteria [3], the patient was identified as having possible infective endocarditis (one major microbiological criterion; two minor criteria: fever and radiological evidence of septic pulmonary infarcts). As a result, the antibiotic therapy was modified, involving the discontinuation of piperacillin/tazobactam, the initiation of oxacillin, and the continuation of daptomycin.

**Figure 4:**
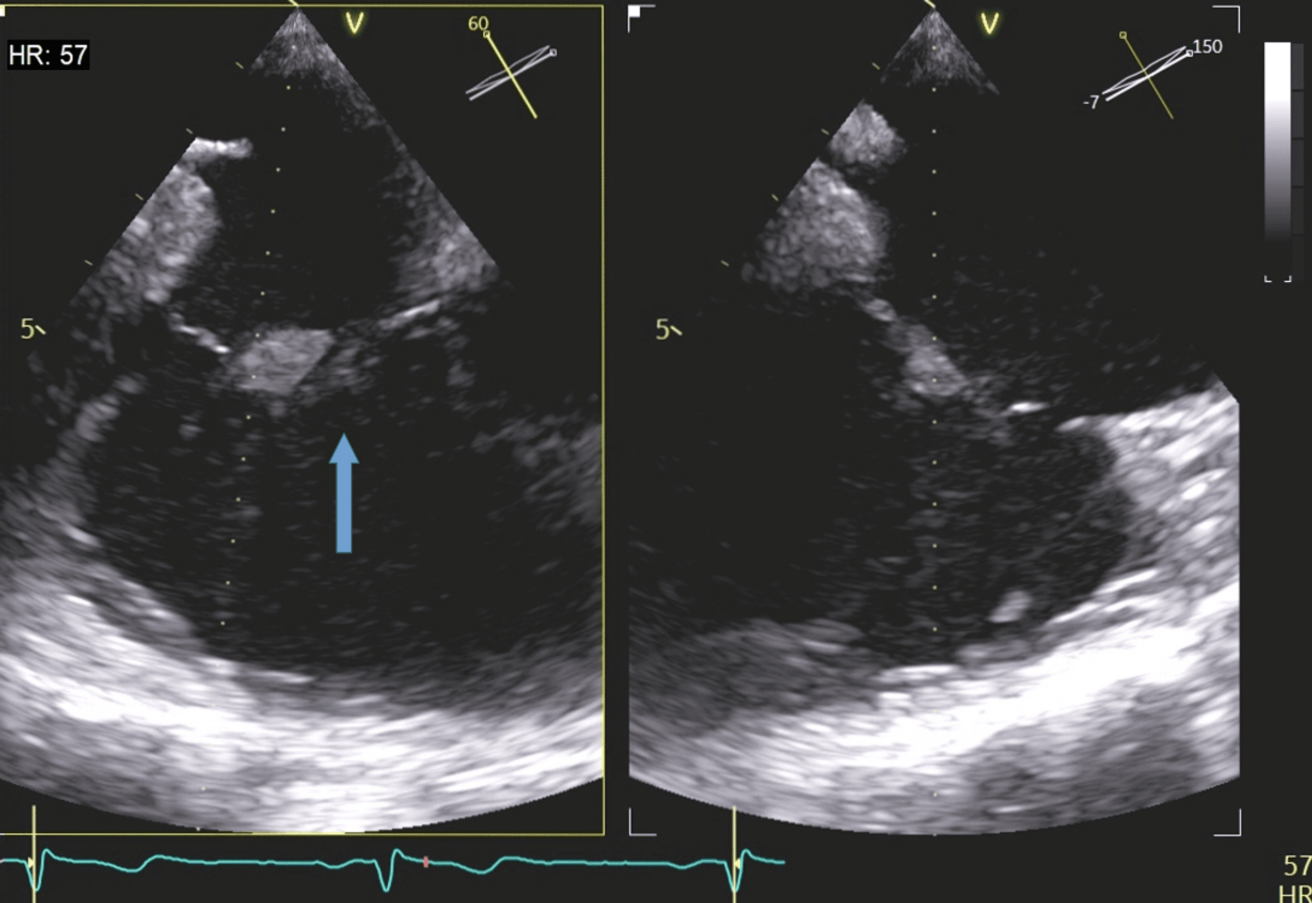
Transesophageal echocardiogram (TE) shows endocarditis of the native tricuspid valve

Confirmatory testing was ordered. A transesophageal echocardiogram (TE) was consistent with infective endocarditis of the native tricuspid valve, with no involvement of the other valves (Figure 4). Subsequent ophthalmic examination ruled out ocular involvement. It also showed no signs of diabetic retinopathy. CT scans of the brain and spine were also negative, while kidney and liver septic emboli were detected by a CT of the abdomen.

Follow-up TE showed a new vegetation involving the pulmonary valve. Surgical valve replacement was not indicated due to the small vegetation sizes.

**Figure 5:**
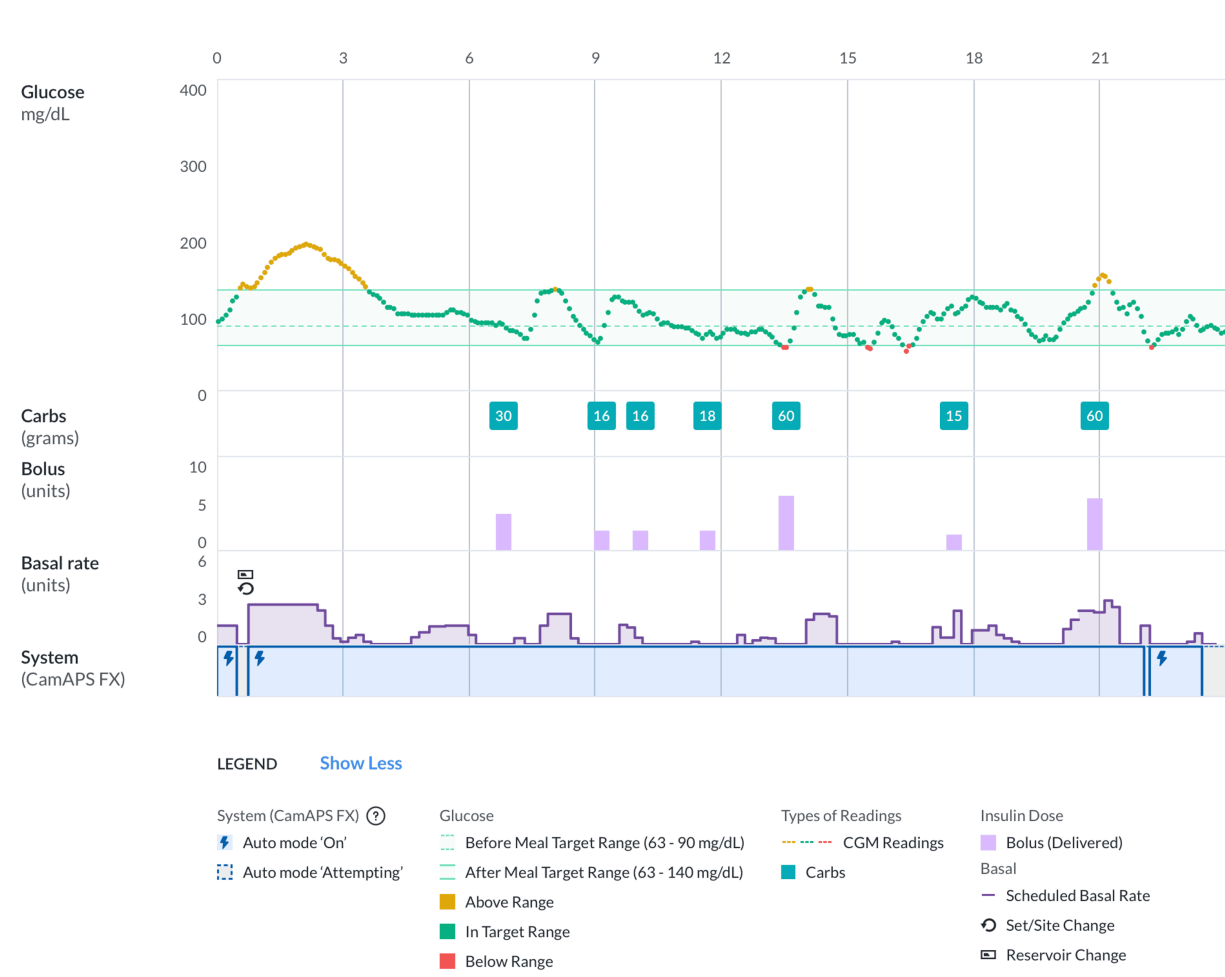
Antibiotic therapy was administered in 1 L 5% dextrose solution CGM: continuous glucose monitoring

Throughout the hospitalization, the patient always remained conscious and capable of self-managing her devices, and therefore, she kept using the CamAPS algorithm with close supervision by our diabetes service. The administration of intravenous antibiotic therapy occurred in a 1 L 5% dextrose solution over four hours, providing 50 g of CHO. This could have posed a further challenge in glucose management. The strategy we used involved the administration of three divided insulin boluses, with an input of 16 g of CHO each for the first two boluses and 18 g for the third bolus (Figure 5).

During hospitalization, the CamAPS settings were adapted following frequent consultations with the diabetes team. The glycemic target was set between 90 and 100 mg/dL, the insulin sensitivity factor was at 70 mg/dL per unit, and IC ratios were 1:8 for breakfast, 1:9.5 for lunch, and 1:11 for dinner. The patient maintained a very good glycemic control despite the ongoing severe infection and the intravenous dextrose solution, with a TIR 70-180 mg/dL of 88%, TAR of 7 + 1%, TBR of 4%, CV of 32.6, and mean glucose of 118 mg/dL. Infective endocarditis resolved within three months without any sequelae.

## Discussion

The advantages of advanced insulin delivery (AID) systems during pregnancy are well established: these systems can help to achieve near-desired glycemic targets while minimizing hypoglycemia in pregnant women with type 1 diabetes [4,5]. Despite the recent findings from trials such as AiDAPT and CRISTAL [5,6], the postpartum period still represents a difficult period to control.

This case report describes the clinical experience of a patient with type 1 diabetes during pregnancy and in the postpartum period, managed with the support of the CamAPS FX hybrid closed-loop algorithm. In the postpartum period, the management of the mother is complicated by physiological and lifestyle changes associated with the care of a newborn, changes in insulin sensitivity, initiation or discontinuation of breastfeeding and possible related problems, and changes in sleep-wake rhythm, in our case also complicated by infection and hospitalization.

Furthermore, the use of AHCL during hospitalization poses additional challenges, necessitating effective self-management on the part of the patient and a positive interaction with the diabetes team. In this setting, the CamAPS FX app facilitated remote and shared therapy management, allowing healthcare professionals to monitor glycemic trends and optimize treatment when necessary. The use of AHCL during hospitalization poses additional challenges, primarily associated with elevated glucose levels caused by the underlying illness and stress, an increased risk of ketoacidosis in the event of pump blockage or malfunction, and the management of the pump during imaging studies.

It has been demonstrated that the use of a hybrid closed-loop system is safe during hospitalization [7]; some authors suggest continuing the closed-loop mode during hospitalization only if the diabetes team is present; otherwise, the AID system should be switched from auto-mode to manual mode (as an insulin pump only) [8]. Some trials have also evaluated the use of a fully closed loop in hospitalized patients with good results [9].

Despite these promising results, most current guidelines do not include specific recommendations regarding the use of AHCL systems in the hospital, although a 2024 guideline from the Association of British Clinical Diabetologists provided some instruction on the use of continuous subcutaneous insulin infusion in hospitalized patients [10]. In this context, we observed, for the first time in this setting, that the use of the CamAPS FX system could be particularly safe in the management of type 1 diabetes even during acute illness. We indeed observed that despite the acute illness, the need for prolonged hospitalization, the antibiotic therapy, the irregular diet, and the postpartum period, the system proved to be effective in reducing nocturnal hypoglycemia (particularly common postpartum), maintaining a satisfactory TIR. Another important point in our case that should be emphasized is that the use of the AHCL system reduced the patient's decision-making burden and stress, which is crucial when physical and psychological demands are considerable such as postpartum and during acute illness. This observation is consistent with what is reported in the literature on the positive impact of closed-loop systems on therapeutic satisfaction and the reduction of diabetes-related stress [11,12].

Notwithstanding the encouraging outcomes, it is imperative to underscore that the implementation of AID systems during pregnancy and the postpartum period necessitates that the patient be thoroughly educated on their utilization and be provided with comprehensive support from the care team. A primary focus must be placed on the consideration of the fluctuation in insulin sensitivity, which can vary considerably among individuals and over the course of time. Additionally, it is crucial to assess the interplay between dietary intake and insulin requirements in the context of breastfeeding. Furthermore, the patient's capacity to operate the device during a period that is both physically and cognitively demanding must be taken into account.

The present case is of particular interest and challenge, due to the occurrence of an early infectious complication following delivery and during breastfeeding, which resulted in hospitalization. Moreover, the administration of dextrose solution for the endovenous (EV) delivery of antibiotic therapy could have further elevated glucose levels.

## Conclusions

The CamAPS FX system has proven to be effective, safe, and adaptable to achieve good glycemic control and reduce the therapeutic burden on women during such a vulnerable period. The use of CamAPS FX in the management of pregnancy and hospitalization in type 1 diabetes shows promising benefits, particularly in improving glycemic control and quality of life. However, patients remain very important. Further studies and trials are needed to confirm the long-term safety and efficacy of hybrid closed-loop systems in postpartum care and to explore how to best integrate them into routine clinical practice, and define shared protocols and guidelines for the use of these technologies in the postpartum period and during hospitalization.
